# Comparison of Masimo O3 and INVOS 7100 cerebral oxygenation during immediate neonatal transition

**DOI:** 10.1007/s00431-026-07017-y

**Published:** 2026-05-19

**Authors:** Prathomwalee Sintupech, Amonrat Anantho, Kamonrat Songnok, Ratchada Kitsommart

**Affiliations:** 1 Division of Neonatology, Department of Pediatrics, Faculty of Medicine Siriraj Hospital, Mahidol University, 2 Wanglang Road, Bangkok Noi, Bangkok, 10700 Thailand; 2 Nursing Division, Department of Obstetrics and Gynaecology, Faculty of Medicine Siriraj Hospital, Mahidol University, Bangkok, Thailand

**Keywords:** Cerebral oxygenation, INVOS 7100, Masimo O3, Near-infrared spectroscopy, Neonatal transition

## Abstract

This study aims to compare CrSO_2_ measured simultaneously by Masimo O3 and INVOS during the first 15 min after birth in healthy term infants and to evaluate agreement and derived cerebral fractional tissue oxygen extraction (cFTOE). In this prospective observational study, 65 healthy term infants were monitored immediately after birth using Masimo O3 and INVOS 7100 applied simultaneously with randomized hemispheric placement. Pre-ductal oxygen saturation (SpO_2_) was recorded continuously. Minute-specific values were derived from aggregated measurements during the final 30 s of each minute. Paired comparisons were performed using Wilcoxon signed-rank test. Agreement was assessed using Bland–Altman analysis with regression evaluation for proportional bias. Both devices demonstrated a similar temporal increase in CrSO_2_ during the first 5 min, with relative stabilization thereafter. From minute 4 onward, Masimo O3 values were lower than INVOS, with significant differences at minutes 5 to 11 and min 14 (*p* < 0.05). Overall mean bias was − 2.8%, with 95% limits of agreement from + 18.0 to − 23.6%. Significant proportional bias was present (*R*^2^ = 0.11, *p* < 0.001). When CrSO_2_ was < 60%, mean bias was + 28.7% with wide limits of agreement. Masimo O3 derived cFTOE values were consistently higher at several time points. No significant hemispheric differences were detected.

*Conclusion*: Masimo O3 and INVOS show similar temporal patterns but an oxygenation-dependent proportional bias. CrSO_2_ values are device-specific and not interchangeable, particularly at lower saturation levels during early transition.

**Table:** 

**What is Known:** • *Cerebral oxygenation during neonatal transition increases rapidly after birth and can be monitored using near-infrared spectroscopy.* • *Absolute values differ between devices, limiting comparability across NIRS systems.* **What is New:** • *This study provides a simultaneous head-to-head comparison of Masimo O3 and INVOS during immediate neonataltransition, demonstrating oxygenation-dependent proportional bias.* • *CrSO2 values are device-specific and and should not be directly translated between monitoring platforms.*

**Supplementary information:**

The online version contains supplementary material available at 10.1007/s00431-026-07017-y.

## Introduction

The immediate neonatal transition represents the complex and rapid physiologic adaptations [1, 2]. Caregivers must understand these physiological adaptations and allow this transition period to proceed normally while remaining vigilant for signs of compromise.

Current Neonatal Resuscitation Program (NRP) guidelines emphasize monitoring arterial oxygen saturation measured by pulse oximetry (SpO_2_) to guide oxygen supplementation after birth [3]. However, it is challenging to rely solely on SpO_2_ to guide oxygen supplementation during birth resuscitation, particularly in infants with abnormalities affecting other components of oxygen content, such as cardiac output, hemoglobin concentration, or regional blood flow distribution [4]. The neonatal brain is characterized by high oxygen consumption and limited tolerance for both hypoxia and hyperoxia [5–7]. Therefore, reliance solely on SpO_2_ may not fully capture cerebral oxygen balance during immediate transition.

There is growing interest in using near-infrared spectroscopy (NIRS) to monitor regional tissue oxygen saturation during the neonatal transition, particularly in high-risk infants [8]. Several studies have described the evolution of cerebral regional oxygen saturation (CrSO_2_) during neonatal transition [9–11]. A recent systematic review demonstrated that CrSO_2_ values are initially low immediately after birth and progressively increase over the first minutes of life, although the pattern differs somewhat from SpO_2_ [8].

Despite growing interest in cerebral monitoring, CrSO_2_ values are highly device dependent. Differences in emitted wavelengths, sensor geometry, assumed arterial to venous blood ratios, signal processing algorithms, and artifact rejection mechanisms produce systematic and oxygenation-dependent bias. Consequently, absolute values obtained from different devices cannot be used interchangeably [12]. Historically, much of the normative data during immediate transition has been generated using the INVOS platform [13]. However, technological advances have introduced newer devices incorporating multiple wavelengths and enhanced signal processing. Without direct comparison and device-specific reference data, applying historical thresholds to newer devices may lead to inaccurate interpretation and potentially inappropriate intervention.

The Masimo O3 system is one such device that provides neonatal sensors and has been increasingly adopted in clinical practice. It utilizes four wavelengths and assumes a 30:70 arterial-to-venous ratio [14], whereas the INVOS employs two wavelengths and assumes a 25:75 ratio [15]. These differences in optical design and algorithmic modeling may influence both absolute values and dynamic response during rapid physiological change [16, 17]. Although previous studies have suggested device-dependent discrepancies during neonatal transition [18, 19], simultaneous head-to-head comparison between Masimo O3 and INVOS during the immediate postnatal period remains limited. Given that the first 15 min after birth represent the period of greatest physiological instability, understanding device behavior during this window is clinically essential.

The primary objective of this study was therefore to compare CrSO_2_ measured simultaneously by Masimo O3 and INVOS during the first 15 min after birth in healthy term infants. Secondary objectives were to characterize cerebral fractional tissue oxygen extraction (cFTOE) and to examine potential hemispheric differences. We hypothesized that CrSO_2_ values measured by Masimo O3 would differ systematically and proportionally from those measured by INVOS during immediate neonatal transition.

## Material and methods

This prospective observational study was conducted in the delivery room at the Department of Obstetrics and Gynecology, Siriraj Hospital, Mahidol University, Bangkok, Thailand. The study protocol was approved by the Siriraj Institutional Review Board (COA no. Si 945/2024), and written informed consent was obtained from parents prior to delivery.

Term neonates (gestational age ≥ 37 weeks) who were vigorous at birth were eligible for inclusion. Infants were excluded if they had major congenital malformations, central nervous system abnormalities, critical congenital heart disease, or scalp abnormalities that could interfere with sensor placement. To ensure analysis of physiological transition rather than pathologic adaptation, infants requiring supplemental oxygen, positive pressure ventilation, or continuous positive airway pressure during the 15-min monitoring period were excluded from the final dataset.

The time of birth was identified as complete delivery of the infant’s body from the birth canal. Immediately after complete delivery of the infant, NIRS sensors from both devices were applied simultaneously to the left and right frontoparietal regions. Sensor allocation to each hemisphere was determined by blocked randomization, with a block size of 4, using sealed opaque envelopes to minimize systematic hemispheric bias. Sensors were positioned approximately one to two centimeters above the eyebrow and lateral to the midline and secured carefully with cohesive bandage to minimize displacement.

The Masimo O3 regional oximeter (Masimo Corporation, Irvine, CA, USA) connected to the Masimo ROOT monitoring platform was used with neonatal sensors. This device utilizes four wavelengths and assumes a 30:70 arterial-to-venous blood volume ratio, recording data every 2 s. The INVOS 7100 cerebral oximeter (Medtronic, Minneapolis, MN, USA) was used with neonatal sensors and employs two wavelengths with an assumed 25:75 arterial-to-venous ratio, recording data every 5 s. Peripheral arterial oxygen saturation (SpO_2_) was monitored preductally using the Masimo ROOT® system with Masimo SET® pulse oximetry technology with a sensor placed on the right wrist, with data recorded every 2 s. Internal clocks of both NIRS devices were synchronized within 1 s prior to each monitoring session to ensure temporal alignment. Monitoring commenced immediately after birth and continued uninterrupted for 15 min. During this period, infants received standard care according to the NRP 8th edition guidelines [20]. Data were recorded continuously and subsequently exported for offline analysis.

To reduce transient artifact and emphasize physiological trend rather than instantaneous fluctuation, the value representing each minute was calculated from aggregated measurements during the final 30 s of that minute. Cerebral fractional tissue oxygen extraction (cFTOE) was calculated for each time point using the formula cFTOE = (SpO_2_ − CrSO_2_)/SpO_2_.

### Sample size calculation

Sample size estimation was based on previously reported paired differences in CrSO_2_ between devices during neonatal transition [18, 19]. Assuming a two-sided alpha level of 0.05 and statistical power of 0.90 for paired comparison, a minimum of 58 infants were required to detect a clinically meaningful difference. To account for potential data loss due to sensor displacement or post enrollment exclusion, an additional 10 percent was added, resulting in a target enrollment of 65 infants. Sample size calculation was performed using G*Power software version 3.1.9.7.

### Statistical analysis

Continuous variables were assessed for distribution and presented as mean ± standard deviation (SD) or median with 25th and 75th percentiles [P25, P75] as appropriate. Categorical variables were expressed as number and percentage. Paired minute-by-minute comparisons between devices were performed using the Wilcoxon signed rank test. We compared baseline characteristics of infants randomized to different sensor placement using chi-square test or Fisher’s exact test for categorical variables and independent-samples *t*-test or Mann–Whitney *U* test for continuous variables, as appropriate.

Agreement between devices was evaluated using Pearson correlation coefficient (*r*) to assess the linear association between paired measurements, followed by Bland–Altman analysis to assess agreement between Masimo O3 and INVOS 7100. To directly compare the readings, we used the value corresponding to that minute, rather than the aggregated data. Bias was defined as the mean difference between Masimo O3 and INVOS values. Limits of agreement were calculated as bias ± 1.96 SD of the differences. To evaluate proportional bias, linear regression analysis was performed with the difference plotted against the average of the two devices. A two-sided *p* value < 0.05 was considered statistically significant. Statistical analyses were performed using IBM SPSS Statistics (IBM Corp., Armonk, NY, USA) version 27. Graphical time course representations were generated using R statistical software.

## Results

Sixty-five healthy term infants were enrolled between 5th February and 21 st November 2025. All infants were singletons and received deferred cord clamping for at least 30 s. Median 1-min Apgar scores were 8 [8, 9] in both groups. None required respiratory support during the monitoring period. Four infants (6.2%) were classified as small-for-gestational age without abnormal antenatal doppler findings. Baseline characteristics were comparable regardless of sensor placement side (Table 1 and Supplementary Table 1).

**Table 1:** Infant demographic characteristics (*N* = 65)

	Total *N* = 65	Masimo O3		p*
		Left *n* = 33	Right *n *= 32	
Gestational age (week)	39 [38.0, 39.0]	39 [38, 39]	38 [37.3, 39]	0.28
Male sex	28 (43.1)	15 (45.5)	13 (40.6)	0.69
Birth weight (g)	3084.2 ± 314.7	3131.5 ± 290.0	3035.3 ± 335.9	0.22
Length (cm)	50 [49, 51]	50 [48.5, 51]	49.5 [49, 51]	0.83
Head circumference (cm)	33 [33, 34]	33 [33, 34.3]	33 [33, 34]	0.78
Small-for-gestational age	4 (6.2)	1 (3)	3 (9.4)	0.36
5-min Apgar scores	9 [9, 10]	9 [9, 10]	9.5 [9, 10]	0.54

Data are presented as number (%), mean ± standard deviation, or median [25th, 75th percentile]

*P*-values were calculated to compare variables between infants who had the Masimo O3 sensor placed on the right frontoparietal area and those on the left side, with *p < 0.05 denoting statistical significance

**Fig. 1:**
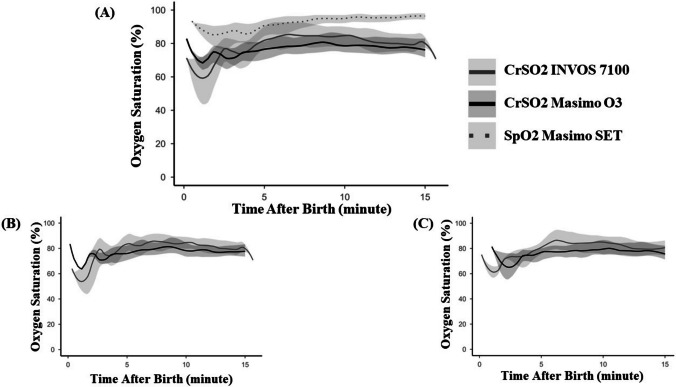
Temporal changes in cerebral oxygen saturation and pre-ductal arterial oxygen saturation during the first 15 min after birth. Plots display median values with interquartile ranges [25th, 75th percentile] as shaded areas. **A** Overall data. **B** Measurements from the right frontoparietal region. **C** Measurements from the left frontoparietal region. Abbreviations: CrSO2, cerebral oxygen saturation, SpO2, peripheral arterial oxygenation

The temporal progression of cerebral regional oxygen saturation (CrSO_2_) measured by Masimo O3 and INVOS during the first 15 min after birth is presented in Fig. 1A, together with SpO_2_ measured by pulse oximetry. For both devices, CrSO_2_ values were low during the first minutes after birth, reached their lowest median value at minute 3, and subsequently increased over time. After minute 5, the increase became less steep and values demonstrated relative stabilization. The pattern of increase in CrSO_2_ occurred in parallel with the progressive rise in SpO_2_ during the same time interval. When analyzed according to sensor placement side, the temporal trajectories of CrSO_2_ measured by Masimo O3 and INVOS showed a consistent pattern on both the right and left frontoparietal regions (Fig. 1B and C). The relative difference between devices across time was similar irrespective of hemispheric location.

Minute-by-minute comparisons of CrSO_2_ and cFTOE are shown in Table 2. At minute 1 and minute 3, the median CrSO_2_ measured by Masimo O3 was slightly higher than that measured by INVOS. However, these differences were not statistically significant. From minute 4 onward, CrSO_2_ values recorded by Masimo O3 were lower than those recorded by INVOS for the remainder of the monitoring period. Statistically significant differences between devices were observed at 5 through 11 min and at minute 14, with *p* < 0.05. Across the 15-min monitoring period, cFTOE values calculated using Masimo O3 measurements were numerically higher than those calculated using INVOS values at most time points. Statistically significant differences in cFTOE were identified from 7 through 11 min and at minute 14 (*p* < 0.05).

**Table 2:** Minute-by-minute comparison of cerebral oxygen saturation and cerebral fractional tissue oxygen extraction measured by Masimo O3 and INVOS during the first 15 min after birth in healthy term infants (*N* = 65)

Time of birth (minute)	CrSO_2_ (%)			cFTOE		
	Masimo O3	INVOS	p*	Masimo O3	INVOS	p*
1	77.0 [50.8, -]	63.7 [29.3, 76.8]	0.18	0.18 [0.18, 0.18]	0.25 [0.07, -]	-
2	74.5 [61.5, 79.5]	79.2 [65.9, 84.8]	0.87	0.15 [0.00, 0.17]	0.12 [0.02, 0.30]	0.69
3	72.0 [67.0, 78.5]	69.5 [63.0, 82.0]	0.39	0.15 [0.09, 0.20]	0.15 [0.08, 0.22]	0.35
4	74.7 [68.6, 81.3]	76.0 [68.0, 81.2]	0.93	0.16 [0.09, 0.22]	0.10 [0.05, 0.23]	0.78
5	76.9 [70.4, 82.7]	80.8 [73.5, 87.8]	0.001*	0.15 [0.09, 0.20]	0.11 [0.03, 0.16]	0.01
6	76.8 [73.3, 85.5]	85.5 [76.0, 91.2]	0.003*	0.13 [0.07, 0.19]	0.10 [0.03, 0.16]	0.08
7	78.0 [72.9, 84.3]	84.3 [78.1, 93.7]	< 0.001*	0.13 [0.08, 0.18]	0.08 [0.02, 0.15]	< 0.001*
8	80.0 [74.6, 84.8]	84.3 [76.6, 92.0]	0.01*	0.14 [0.09, 0.21]	0.11 [0.05, 0.17]	0.02*
9	79.8 [74.3, 86.4]	83.7 [77.8, 90.7]	0.01*	0.15 [0.08, 0.21]	0.11 [0.05, 0.17]	0.004*
10	79.3 [74.6, 85.1]	85.0 [77.9, 89.2]	0.003*	0.14 [0.11, 0.20]	0.10 [0.05, 0.17]	0.003*
11	78.9 [73.6, 83.3]	82.2 [76.8, 89.5]	0.01*	0.16 [0.12, 0.24]	0.13 [0.06, 0.19]	0.01*
12	77.6 [74.3, 84.9]	80.3 [75.3, 87.2]	0.09	0.16 [0.12, 0.23]	0.15 [0.07, 0.20]	0.10
13	77.3 [77.3, 84.0]	80.8 [74.8, 85.5]	0.11	0.18 [0.13, 0.24]	0.16 [0.10, 0.19]	0.05
14	76.9 [73.5, 82.9]	79.2 [76.0, 86.0]	0.04*	0.19 [0.14, 0.24]	0.18 [0.10, 0.21]	0.04*
15	77.0 [72.0, 84.0]	79.0 [74.5, 84.5]	0.10	0.20 [0.14, 0.26]	0.18 [0.12, 0.22]	0.14

Data presents in median [25th, 75th percentile]. *p-value < 0.05 is considered statistically significant

*CrSO*_*2*_ cerebral oxygen saturation, *cFTOE* cerebral fractional tissue oxygen extraction

**Fig. 2:**
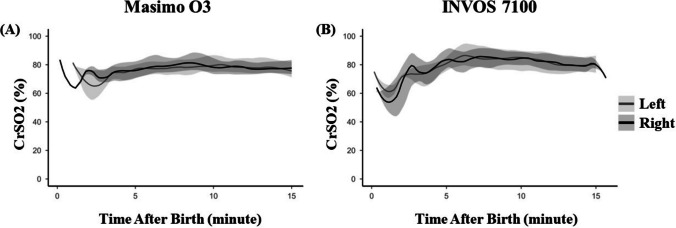
Comparison of left and right hemispheric cerebral oxygen saturation during the first 15 min after birth. Plots display median values with interquartile ranges [25th, 75th percentile] displayed as shaded areas. **A** CrSO_2_ measurements from Masimo O3 device. **B** Measurements from INVOS device. Abbreviations: CrSO2, cerebral oxygen saturation

Supplementary Table 2 presents side-specific comparisons between devices at each minute. On the right side, significant differences in CrSO_2_ between Masimo O3 and INVOS were observed at minutes 7, 10, and 11. On the left side, significant differences were identified at minutes 6 and 7. At all other time points, differences did not reach statistical significance. Figure 2 displays CrSO_2_ values measured on the left and right cerebral hemispheres for each device separately. For both Masimo O3 and INVOS, the temporal trajectories of left and right hemispheric CrSO_2_ were closely aligned throughout the 15-min monitoring period. Interquartile ranges overlapped at all time points. Statistical analysis demonstrated no significant differences between left and right hemispheric measurements for either device at any minute, as shown in Supplementary Table 3.

**Fig. 3:**
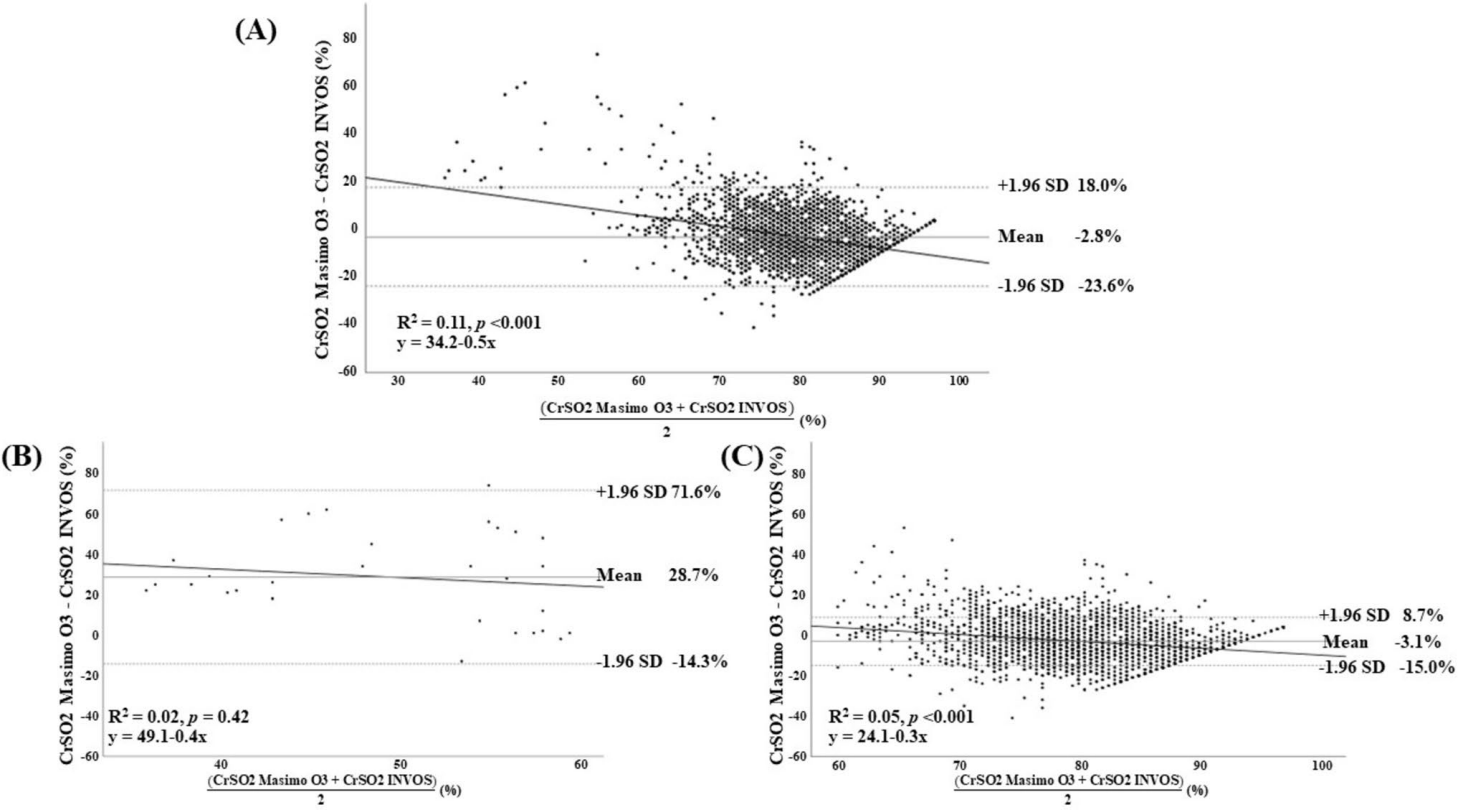
Bland-Altman plots comparing cerebral oxygen saturation measured by Masimo O3 and INVOS. **A** All paired measurements. **B** Values < 60%, **C** values ≥ 60%. Bias is defined as Masimo O3 minus INVOS. The solid line indicates mean bias and dashed lines indicate 95% limits of agreement. The regression line demonstrates proportional bias

Pearson correlation analysis demonstrated a positive correlation between CrSO_2_ measured by Masimo O3 and INVOS 7100 (*r* = 0.35, *p* < 0.001). Agreement between devices was further assessed using Bland–Altman analysis (Fig. 3). The overall mean bias, defined as CrSO_2_ Masimo O3 minus CrSO_2_ INVOS, was − 2.8%. The 95% limits of agreement ranged from + 18.0 to − 23.6%, corresponding to a total span of 41.6%. Linear regression analysis of the differences plotted against the averages demonstrated a statistically significant slope (*y* = 34.2 − 0.5*x*; *R*^2^ = 0.11; *p* < 0.001), indicating the presence of proportional bias.

When analysis was restricted to paired measurements in which CrSO_2_ values from both devices were below 60% (Fig. 3B), the mean bias was + 28.7%, with limits of agreement ranging from + 71.6 to − 14.3%. In the subgroup of paired measurements with CrSO_2_ values exceeding 60% (Fig. 3C), the mean bias was − 3.1%, with limits of agreement from + 8.7 to − 15.0%. Proportional bias remained statistically significant in this range (*p* < 0.001).

## Discussion

In this prospective head-to-head comparison of two commercially available NIRS devices during immediate neonatal transition, we observed that Masimo O3 and INVOS demonstrated similar temporal trends in CrSO_2_ during the first 15 min after birth. However, absolute values differed in a proportional manner across the oxygenation spectrum, with oxygenation-dependent divergence particularly evident at lower CrSO_2_ levels.

The physiological trajectory observed in both devices was consistent with established knowledge of neonatal transition [8, 18]. CrSO_2_ values were lowest during the early minutes after birth and increased progressively. This pattern paralleled the rise in SpO_2_ and is in agreement with previous observational studies [13, 21, 22]. The similarity in temporal pattern between devices supports the ability of both systems to capture dynamic changes during physiological adaptation [8, 11].

Although only a weak-to-moderate positive correlation was observed between the two devices (*r* = 0.35), agreement analysis demonstrated that the inter-device difference was not characterized by a constant systematic offset but rather by proportional bias [10, 12]. The regression slope in the Bland–Altman analysis was statistically significant, indicating that both the magnitude and direction of inter-device difference depended on the level of oxygenation. When CrSO_2_ values were below 60%, Masimo O3 demonstrated substantial positive bias relative to INVOS. At higher saturation levels, the bias shifted in the opposite direction. This distinction between systematic and proportional bias is clinically important. Several previous studies assumed systematic bias between devices [19, 23, 24]. A purely systematic bias implies a constant offset that might theoretically allow adjustment using a simple correction factor. In contrast, proportional bias indicates that the discrepancy varies across the measurement range. Our findings therefore support prior evidence suggesting that cross-device conversion equations are unreliable and reinforce the concept that absolute CrSO_2_ values are device-specific [11].

The wider limits of agreement observed at lower CrSO_2_ levels deserve particular attention. The early minutes after birth are characterized by rapid changes in pulmonary vascular resistance, cardiac preload and afterload, and cerebral blood flow [1, 2]. During this phase, cerebral oxygenation reflects the interaction between oxygen delivery and metabolic extraction. Given the venous-weighted nature of NIRS measurement [15], differences in assumed arterial to venous ratios, wavelength selection, and signal processing algorithms may contribute to the observed inter-device differences, particularly when oxygen saturation is low and cerebral extraction is high.

We also observed that CrSO_2_ measured by Masimo O3 demonstrated less fluctuation during the earliest minutes compared with INVOS, which showed greater variability. A similar phenomenon has been described in pulse oximetry under hypoxic conditions, where device-specific signal processing and averaging algorithms influence variability [25, 26]. Although the present study was not designed to directly assess precision, the observed pattern is consistent with prior reports describing device-dependent performance characteristics in NIRS systems [9, 27].

Since cFTOE incorporates information from systemic arterial oxygenation and regional cerebral oxygenation simultaneously, it tends to demonstrate greater stability than CrSO_2_ alone during transition and may provide a more integrated representation of cerebral oxygen balance [27]. In our cohort, Masimo O3 derived cFTOE values were consistently higher and demonstrated a narrower range compared with INVOS. Previous work has shown that both respiratory and hemodynamic conditions significantly influence cFTOE during transition [28]. These findings indicate that cFTOE, similar to CrSO_2_, is dependent on device-specific measurement characteristics and should not be interpreted independently of the monitoring platform used.

CrSO_2_ values in our cohort, particularly those measured by Masimo O3, were generally higher than the reported minute-specific medians [8]. Methodological differences may partly explain this observation. In our study, values were derived from aggregated measurements during the final 30 s of each minute, which reduces transient fluctuation and may produce slightly higher stable values. In addition, although the meta-analysis included both term and preterm infants, most preterm infants were late preterm, and previous studies have shown minimal difference between term and late-preterm CrSO_2_ during transition [29, 30]. These comparisons highlight the importance of considering both device type and analytic methodology when interpreting published reference ranges.

We found no significant difference between left and right hemispheric measurements in stable term infants. Previous reports have described minor hemispheric variability, particularly under unstable systemic oxygenation [31]. However, other investigations have demonstrated minimal difference in stable neonates [32, 33]. Our findings support the use of unilateral monitoring in stable term infants and suggest that hemispheric placement does not substantially influence interpretation during physiological transition.

From a clinical perspective, our data suggest caution in defining rigid universal CrSO_2_ targets during the earliest minutes after birth. Unlike SpO_2_, for which reference curves have been incorporated into the NRP guidelines [3], CrSO_2_ lacks standardized device-independent thresholds. The wide limits of agreement and proportional bias observed at lower saturation levels indicate that minute-specific CrSO_2_ targets analogous to SpO_2_ nomograms may not be appropriate across devices, particularly during the first 5 min. Monitoring trends during this early phase may provide more reliable information than relying on absolute numeric thresholds. However, inter-device differences become clinically relevant when absolute CrSO_2_ values are interpreted against reference ranges or used to guide clinical decision-making. After minute 5, when CrSO_2_ values stabilized within the commonly reported physiological range of approximately 60% to 85% [10, 27], inter-device differences were smaller relative to the overall range, suggesting that device-specific normative targets may be more applicable during this later phase.

The strengths of this study include simultaneous measurement using two widely utilized devices during the most dynamic period of neonatal transition, rigorous synchronization, randomized hemispheric placement, and analysis across both low and high CrSO_2_ ranges. Importantly, we demonstrate proportional rather than purely systematic bias, contributing meaningful evidence to the existing literature on device comparability. One limitation of this study is that the cohort consisted exclusively of healthy term infants. Therefore, extrapolation of absolute CrSO_2_ and cFTOE reference values to preterm or high-risk populations should be performed with caution, as differences in cerebral autoregulation, transitional hemodynamics, and oxygen extraction may influence physiological patterns in those groups. However, the primary aim of this study was to compare simultaneous numerical readings between two monitoring systems. The observed proportional and systematic differences reflect device-specific optical configuration and algorithmic processing rather than gestational age–dependent physiology. As such, the inter-device relationship identified in this study is likely applicable across gestational ages, even though absolute normative values may differ in preterm or compromised infants. Importantly, our analysis included measurements across both lower and higher CrSO_2_ ranges encountered during immediate transition, strengthening the validity of the device comparison throughout the physiological spectrum. Overall, our findings reinforce that CrSO_2_ values are devices-specific and not interchangeable. Interpretation must consider the specific monitoring system used, and direct translation of reference ranges between devices is not supported by our data.

In conclusion, Masimo O3 and INVOS demonstrated similar temporal trends in CrSO_2_ during the first 15 min after birth in healthy term infants. However, absolute values differed in an oxygenation-dependent manner, with significant proportional bias, particularly at lower saturation levels. These findings indicate that CrSO_2_ values are device-specific and not interchangeable. During early neonatal transition, interpretation should emphasize trends rather than fixed numeric thresholds, and device-specific validation is essential for clinical application.

## Supplementary information

Below is the link to the electronic supplementary material.

ESM 1 (DOCX 15.9 KB)

ESM 2 (DOCX 17.8 KB)

ESM 3 (DOCX 17.6 KB)

## Data Availability

The datasets generated and/or analyzed during the current study are available from the corresponding author on reasonable request.
