# Supplementary material for: Comparison of Masimo O3 and INVOS 7100 cerebral oxygenation during immediate neonatal transition
Source: Eur J Pediatr. 2026 May 19;185(6):414. doi: 10.1007/s00431-026-07017-y (PMC13190425; doi:10.1007/s00431-026-07017-y)
Supplement: Supplementary file 1 — (DOCX 15.9 KB) [file 431_2026_7017_MOESM1_ESM.docx]

| Supplementary Table 1 Maternal demographic characteristics (N=65) | | | | | |
| --- | --- | --- | --- | --- | --- |
|  | | **Total**  **N=65** | **Masimo O3** | | *p** |
|  |  |  | **Left**  **n=33** | **Right**  **n=32** |  |
| Maternal age (year) | | 29.0±5.2 | 28.7±5.1 | 29.4±5.4 | 0.55 |
| Maternal underlying diseases | |  |  |  |  |
|  | Diabetes | 9 (13.8) | 6 (18.2) | 3 (9.4) | 0.30 |
|  | Hypertension-related disorders | 6 (9.2) | 2 (6.2) | 4 (12.5) | 0.37 |
|  | Thalassemia | 13 (20) | 7 (21.2) | 6 (18.8) | 0.80 |
| Postpartum hemorrhage | | 2 (3.1) | 2 (6.1) | 0 | 0.16 |
| Data are presented as mean ± standard deviation or number (%).  P-values were calculated to compare variables between infants who had the Masimo O3 sensor placed on the right frontoparietal area versus those on the left side, with **p* < 0.05 denoting statistical significance. | | | | | |
