# Supplementary material for: Comparison of Masimo O3 and INVOS 7100 cerebral oxygenation during immediate neonatal transition
Source: Eur J Pediatr. 2026 May 19;185(6):414. doi: 10.1007/s00431-026-07017-y (PMC13190425; doi:10.1007/s00431-026-07017-y)
Supplement: Supplementary file 3 — (DOCX 17.6 KB) [file 431_2026_7017_MOESM3_ESM.docx]

| **Supplementary Table 3 Minute-by-minute comparison of CrSO2 between right and left frontoparietal regions for Masimo O3 and INVOS 7100 separately (N = 65)** | | | | | | |
| --- | --- | --- | --- | --- | --- | --- |
| **Time**  **of birth (minute)** | **CrSO2 Masimo O3 (%)** | | | **CrSO2 INVOS 7100 (%)** | | |
|  | **Right**  **N=32** | **Left**  **N=33** | ***p**** | **Right**  **N=33** | **Left**  **N=32** | ***p**** |
| 1 | 63.9[50.8, -] | 80.0[80, 80] | 0.22 | 63.7[29.0, -] | 49.8[29.5, -] | 1.00 |
| 2 | 75.1[69.1, 79.4] | 61.6[53.7, 80.8] | 0.39 | 80.0[44.4, 85.4] | 76.3[71.8, 85.3] | 0.83 |
| 3 | 72.0[68.2, 77.6] | 70.9[66.8, 81.5] | 0.83 | 74.6[63.9, 87.3] | 69.2[61.8, 77.2] | 0.42 |
| 4 | 74.1[67.3, 81.4] | 74.7[70.0, 81.3] | 0.82 | 75.9[68.3, 82.5] | 76.2[67.7, 81.2] | 0.98 |
| 5 | 75.7[70.5, 83.3] | 77.5[70.2, 82.4] | 0.86 | 82.3[73.9, 90.0] | 79.8[69.6, 83.3] | 0.34 |
| 6 | 76.3[72.3, 87.3] | 77.2[73.6, 84.4] | 0.63 | 80.3[73.0, 87.8] | 86.2[78.3, 94.9] | 0.12 |
| 7 | 78.8[74.5, 84.6] | 77.9[72.7, 83.9] | 0.49 | 84.0[77.8, 92.5] | 84.3[79.4, 94.1] | 0.79 |
| 8 | 82.0[75.3, 85.5] | 77.4[72.4, 83.3] | 0.19 | 84.5[79.5, 91.7] | 84.0[74.6, 93.4] | 0.72 |
| 9 | 79.5[74.3, 87.5] | 80.1[74.8, 85.9] | 0.86 | 83.7[78.7, 89.4] | 84.3[75.7, 91.5] | 0.91 |
| 10 | 79.3[72.6, 84.2] | 79.8[75.5, 87.2] | 0.28 | 84.8[79.0, 90.8] | 85.5[73.8, 88.9] | 0.82 |
| 11 | 79.1[72.8, 83.3] | 78.9[75.2, 83.4] | 0.55 | 81.4[77.0, 88.3] | 84.0[74.2, 90.3] | 0.96 |
| 12 | 77.3[72.9, 81.4] | 78.4[74.4, 85.3] | 0.52 | 80.3[75.7, 87.7] | 80.4[71.0, 86.6] | 0.33 |
| 13 | 76.7[74.1, 82.8] | 77.5[75.0, 85.9] | 0.40 | 80.9[75.7, 85.7] | 79.8[73.6, 84.8] | 0.41 |
| 14 | 76.8[72.9, 82.8] | 77.0[73.6, 83.6] | 0.69 | 79.0[76.0, 86.0] | 80.0[73.6, 86.5] | 0.83 |
| 15 | 79.0[72.0, 84.0] | 76.0[72.0, 83.0] | 0.57 | 78.0[73.5, 84.0] | 80.5[75.0, 87.5] | 0.21 |

Data presents in median [25^th^, 75^th^ percentile]. * *p*-value < 0.05 is considered statistically significant.

CrSO2; cerebral oxygen saturation.
